# A STATEWIDE PROGRAM FOR EARLY AND ACCURATE DIAGNOSIS OF MEMORY LOSS

**DOI:** 10.1093/geroni/igae098.0423

**Published:** 2024-12-31

**Authors:** Theodore Johnson

**Affiliations:** Emory University, Atlanta, Georgia, United States

## Abstract

Georgia Memory Net (GMN) provides clarity, care, and community connection for Georgians. GMN provides Primary Care Providers the educational resources local infrastructure they need to help Georgians experiencing memory loss or cognitive impairment get timely and accurate diagnoses. Georgia Memory Net also provides initial support and education while linking Georgians dealing with Alzheimer’s and related dementias and their care partners with community resources that can provide ongoing support. Primary Care Providers who have detected memory concerns can refer to one of seven Georgia Memory Net Memory Assessment Clinics or one of the rural telemedicine sites co-located in Department of Public Health clinics. Georgia Memory Net is made possible by a mandate from the Georgia State Legislature as a way to support Georgians all around our state who are dealing with memory issues. GMN is administered by experts at the Goizueta Alzheimer’s Disease Research Center at Emory University.

